# A cross-sectional comparison of the functionality of the short-form FFQ to a 3-day food intake record completed early in the second trimester of pregnancy

**DOI:** 10.1017/S0007114525103966

**Published:** 2025-07-28

**Authors:** Karishma Hosein, Taniya S. Nagpal, Roberta Bgeginski, Harry Prapavessis, Isabelle Giroux, Michelle F. Mottola

**Affiliations:** 1 R. Samuel McLaughlin Foundation – Exercise and Pregnancy Lab, School of Kinesiology, Faculty of Health Sciences, Western University, London, Canada; 2 Faculty of Kinesiology, Sport and Recreation, University of Alberta, Edmonton, Canada; 3 Children’s Health Research Institute, London, Canada; 4 Exercise and Health Promotion Lab, School of Kinesiology, Faculty of Health Sciences, Western University, London, Canada; 5 School of Nutrition Sciences, Faculty of Health Sciences, University of Ottawa, Ottawa, Canada; 6 Department of Anatomy & Cell Biology, Schulich School of Medicine & Dentistry, Western University, London, Canada

**Keywords:** Pregnancy, Nutrition assessment, Nutrition intervention, Behaviour change techniques, Dietary habits

## Abstract

Using a behavioural intervention to target nutrition during pregnancy may be key in meeting recommendations for healthy eating. The aim was to assess the use of a short-term dietary intake measurement tool (3-day food intake record) to infer long-term habitual dietary intake during pregnancy (using a short-form FFQ). A convenience sample (*n* 90) between 12- and 18-weeks’ gestation was recruited from a larger randomised controlled trial for cross-sectional analysis. Participants completed a forty-four-item FFQ and 3-day food intake record. Using the participant food intake record, the investigator blindly completed a second frequency questionnaire. The frequency questionnaires were scored using dietary quality scores (DQS) and compared. Aggregate data were evaluated using a Wilcoxon signed rank test, and individual-level data were evaluated using a Bland–Altman plot. No significant difference was observed in the scores (*Z* = –1·88, *P* = 0·06), with small effect size (*r*= 0·19). The Bland–Altman plot showed that comparing the DQS derived from the two different dietary assessments underestimated scores by a mean difference of 0·4 points (95 % limits of agreement: −3·50 to 4·26). The data points were evenly spread suggesting no systematic variation for over- or underestimation of scores. Minimal difference was observed between the functionality of the two assessment instruments. However, the food intake record can be completed by pregnant individuals to estimate short-term nutrient intake and then scored by the investigator to estimate long-term dietary quality. Combining these two instruments may best capture the most accurate representation of dietary habits over time.

Pregnancy represents a uniquely challenging time to reliably track and assess food intake habits, due to physiological changes, dietary preference fluctuations and conditions brought on by pregnancy, like vomiting or nausea^(1)^. Some of the most used self-report instruments for tracking dietary intake are FFQ and 3-day food intake records (3dFR)^(2,3)^.

In research and clinical practice, FFQ are used to assess habitual food and nutrient intake over a specific period of time^(4)^. The FFQ offers versatility in measurement by asking respondents about both the frequency and portion size of each type of food consumed^(5)^. The Short-Form FFQ (SFFFQ) has been validated as a feasible tool for capturing past long-term (one month) dietary intake habits to score and rank diet quality for adult populations^(6)^.

The 3dFR is a reliable instrument for self-report of short-term dietary intake (including specific nutrient intake) with minimal retrospective memory bias^(3)^. Compared with other food recording tolls, like 24-h recalls, the 3dFR does not need to be conducted as a structured interview led by a trained investigator. Additionally, the 3dFR captures nutrient composition information, timing of food and beverage consumption throughout three consecutive days and portion size. A considerable limitation of the 3dFR, however, is respondent burden (i.e. it is time consuming to complete, and there is investigator burden associated with coding and entering data for analysis).

Though both instruments could be used together to measure short- and long-term nutrition patterns, perhaps one instrument alone would be ideal to capture a cross section of dietary habits while also minimising burden to the user. Typically, in an intervention setting, participants are subject to several dietary assessment instruments, often repeated throughout the course of the study. However, if one instrument is effective at acquiring participant nutrition information (including macro- and micro-nutrient consumption) while minimising bias and participant time spent completing instruments, it would be preferred^(7)^. As such, the aim of the present study was to assess the use of a short-term dietary intake measurement tool to make inferences about long-term habitual dietary intake for pregnant individuals in early second trimester. Two self-reported dietary assessment instruments, validated in the non-pregnant population, 3dFR^(8,9)^ and SFFFQ^(6)^, were used to measure the patterns of food intake for pregnant individuals, with the goal of specifically elucidating the differences, if any, between dietary quality scores (DQS) that were derived from (a) pregnant individuals’ SFFFQ and (b) investigator SFFFQ estimated from pregnant individuals’ 3dFR. It was hypothesised that the DQS calculated from an SFFFQ would reflect the same dietary intake habits as a DQS determined from a SFFFQ completed from a 3dFR.

## Methods

Participants were recruited on a rolling basis as a convenience sample from a larger randomised controlled trial (prospectively registered on Clinicaltrials.gov, identifier: NCT02804061; *n* 142). The study was conducted according to the guidelines laid down in the Declaration of Helsinki, and all procedures involving human participants were approved by the institutional ethics review board (project ID: 108080) and complied with the twenty-two-item STROBE Checklist for cross-sectional observational studies. Written informed consent was obtained from all pregnant participants between 12 and 18 weeks gestation (mean age = 14·9 (sd 2·2) weeks). Participants were asked at baseline before randomisation occurred to complete an SFFFQ and 3dFR, on paper and in-person.

From the larger trial, participants were excluded if they were more than 18 weeks’ gestation, were less than 18 years of age, did not have a singleton pregnancy, had existing diabetes, smoked, exceeded the physical activity guidelines during pregnancy based on the PARmed-X For Pregnancy^(10)^, had any other chronic conditions or had any contraindications to participation in physical activity or exercise.

Each participant completed a forty-four-item self-reported SFFFQ_1_^(6)^ during their first visit to the lab. To complete the SFFFQ_1,_ participants recalled the frequency of their food and beverage intake over the previous 4 weeks by estimating and recording the frequency of eating one portion of specific foods and beverages in the following food categories: fruit, juice, salad, vegetables, chips, beans, fibre, wholemeal breads, cheese, crisps, cakes, cream, fizzy drinks, red meat, white meat, sausages, nuggets, battered fish, oily fish and white fish^(11)^. No contextual details regarding preparation, timing or location of food and beverage consumption were captured.

After completing the SFFFQ_1_, each participant was instructed to complete a self-reported 3dFR^(8)^ in which they accounted for all food, beverages and dietary supplements consumed over a period of three consecutive days (including two weekdays and one weekend day) in the following week. Participants were given a recording form and oral and written instructions to record their food, beverage and supplements as they were consumed during the day, including portion sizes, method of preparation, timing and location of meals and snacks. Portion sizes were estimated by the participant at the time of recording using culturally familiar visual cues, an outline of which was also included on the recording form (pictures and text describing visual cues used to estimate food portion size). The 3dFR was an open-ended dietary intake measurement form, so there was no limit as to how many food, beverage or dietary supplement items could be reported. The completed 3dFR was returned upon the participant’s next visit to the lab, approximately 1 week after the initial visit. The investigator reviewed the completed record with the participant and asked for any missing or necessary additional information pertaining to portions sizes, method of food preparation, brand name or ingredients (for composite foods or recipes) when needed.

Using the participant 3dFR, the investigator blindly completed a second SFFFQ (SFFFQ_2_). To transpose the weekday and weekend consumption from the participant 3dFR to the daily and weekly food intake frequencies of the SFFFQ_2_, the number of servings of each food category represented in the SFFFQ_2_ were counted and summed for each weekday and weekend day. To approximate daily or weekly servings of a food, the number of weekday servings from the 3dFR were multiplied by five, and the number of weekend servings from the 3dFR were multiplied by two. These numbers were added together to get the daily or weekly number of servings for a food category in the SFFFQ_2_.

After the SFFFQ_2_ was generated by the investigator, a DQS_2_ was determined following the methodology of Cleghorn *et al.* ^(6)^ and Wall *et al.* ^(11)^ Using equations developed by Cleghorn *et al.* ^(6)^, the DQS was calculated from the SFFFQ scores. The minimum DQS possible was 5 and the maximum was 15, with a higher score representing a higher quality diet^(6)^.

**Figure 1.**
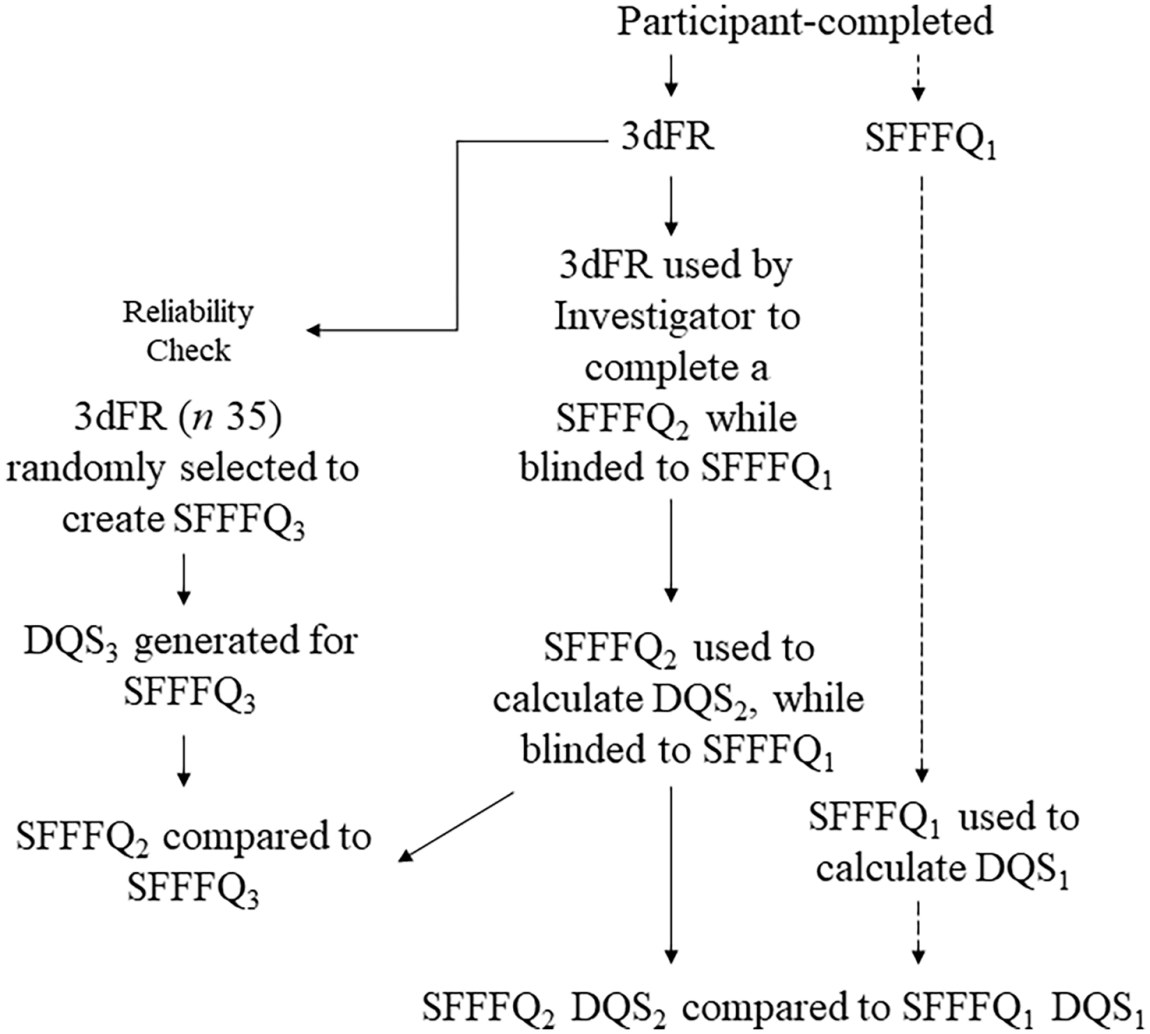
Flow diagram of study methods. 3dFR = 3-day food intake record. SFFFQ = Short-Form FFQ. DQS = Dietary Quality Score. SFFFQ1: Short-Form FFQ completed by participants at recruitment. SFFFQ2: Short-Form FFQ completed by investigator based on 3dFR. SFFFQ3: Short-Form FFQ: completed by the investigator based on 3dFR from a random sample.

A DQS_1_ was determined from the SFFFQ_1_ using the same methods described above. Next, while blinded to the DQS_1_, a DQS_2_ was determined for the SFFFQ_2_ using the same methods described above. The DQS_2_ calculated from the SFFFQ_2_ were then compared to the DQS_1_ generated from the SFFFQ_1_ (Figure 1).

### Statistical analyses

All statistical analyses were performed in IBM Statistical Package for Social Sciences (SPSS) for Windows version 27 (SPSS Inc.). Results were reported as mean (standard deviation) or *n* and %. The significance value was set to *α*< 0·05. Where applicable, effect sizes were calculated using the criteria for interpretation by Cohen^(12)^ where *d*= 0·2 indicates a small effect, *d*= 0·5 indicates a medium effect, and *d* = 0·8 indicates large effect. Descriptive statistics were calculated for participant characteristics. The Shapiro-Wilk’s test was used to test the normality of the SFFFQ score data (DQS). A Wilcoxon signed-rank test was performed to compare DQS_1_ and DQS_2_ of the corresponding SFFFQ_1_ and SFFFQ_2_.

To test the reliability of the scoring system of the SFFFQ_2_ from the 3dFR, an SFFFQ_3_ was developed using the same methods as above for 35 subjects randomly selected from the same 3dFRs (size of reliability sample was calculated *a priori* using G * Power 3, *α* = 0·05, power = 0·8, effect size = 0·5)^(13)^. The SFFFQ_3_ was developed and scored at a different time from the SFFFQ_2_, with the investigator blinded to the DQS_2_ of the SFFFQ_2_ (Figure 1). The two separately generated DQS_2_ and DQS_3_ for each of the analysed 3dFRs were compared using a Student’s paired *t* test to examine the reliability and reproducibility of the scoring system for the SFFFQ_2_.

Limits of agreement between DSQ_1_ and DQS_2_ were determined according to recommendations by Bland and Altman (Bland and Altman, 1986), in which the difference between the scores from the participant generated and investigator generated SFFFQ (DQS_1_–DQS_2_) were plotted against the mean DQS [(DQS_1_ + DQS_2_)/2]. The mean difference (bias) between the two DQS scores and the 95 % limits of agreements were calculated.

## Results

A convenience sample of 90 participants who completed both the SFFFQ_1_ and the 3dFR were included in the current study. Demographic characteristics for all participants (*n* 90) and the sub-sample from the total participants used to check the scoring reliability (*n* 35) are presented in Table 1.

**Table 1.** Descriptive characteristics of participants at the time of completion of dietary intake measurement instruments (Mean values and standard deviations; numbers and percentages)

	All participants (*n* 90)		Reliability sample (*n* 35)	
	Mean	sd	Mean	sd
Gestational age (weeks)	14·9	2·2	15·3	2·1
Maternal age (years)	32·4	3·8	31·5	3·5
Height (m)	1·65	0·07	1·65	0·1
Prepregnancy weight (kg)	70·4	14·5	71·6	15·9
Prepregnancy BMI (kg/m^2^)	25·7	4·8	26·1	5·9
	*n*, %		*n*, %	
BMI category (*n*, %)				
Normal weight	47, 52		21, 60	
Overweight	31, 34		7, 20	
Obese	12, 13		7, 20	
Parity (*n*, %)				
Primiparous	53, 59		23, 66	
Multiparous	37, 41		12, 34	
Ethnicity (*n*, %)				
Caucasian	79, 88		30, 86	
Hispanic	1, 1		–	
Asian	1, 1		–	
Indigenous	2, 2		1, 3	
Mixed	7, 8		4, 11	
Education (*n*, %)				
High School	1, 1		–	
College	12, 13		4, 11	
University (Bachelor)	36, 40		17, 49	
University (Masters)	27, 30		11, 31	
University (Doctorate)	8, 9		3, 9	
Other	5, 6		–	

All data presented as means (standard deviation), unless otherwise indicated.

A Shapiro-Wilk’s test of the DQS_3_ (9·94 (sd 1·85), 95 % CI (9·31, 10·58), *n* 35) and corresponding DQS_2_ (10·37 (sd 1·83), 95 % CI (9·74, 11·00), *n* 35) showed normal distribution (W(35) = 0·96, *P* = 0·31; W(35) = 0·95, *P* = 0·18). A Paired-samples *t* test showed no significant difference in the scores for the DQS_3_ and DQS_2_ (t(35) = –1·04, *P* = 0·30, 95 % CI (–1·26, 0·41)), with a small effect size (η^2^ = 0·03) using Cohen^(12)^ criteria.

Descriptive analysis of the average DQS_1_ (10·62 (sd 1·36), 95 % CI (10·28, 10·9), *n* 90) and DQS_2_ (10·24 (sd 1·52), 95 % CI (9·93, 10·56), *n* 90) were performed. A Shapiro-Wilk’s test showed a significant departure from normality for both DQS_1_ (W(90) = 0·95, *P* = 0·002) and DQS_2_ (W(90) = 0·94, *P* = < 0·001). Due to the nonparametric distribution of data, a Wilcoxon signed rank test was performed, which showed no significant difference in the DQS of the participant-completed SFFFQ_1_ and the investigator-completed SFFFQ_2_ (*Z* = –1·88, *P* = 0·06) with a small effect size (*r* = 0·19) using Cohen^(12)^ criteria.

**Figure 2.**
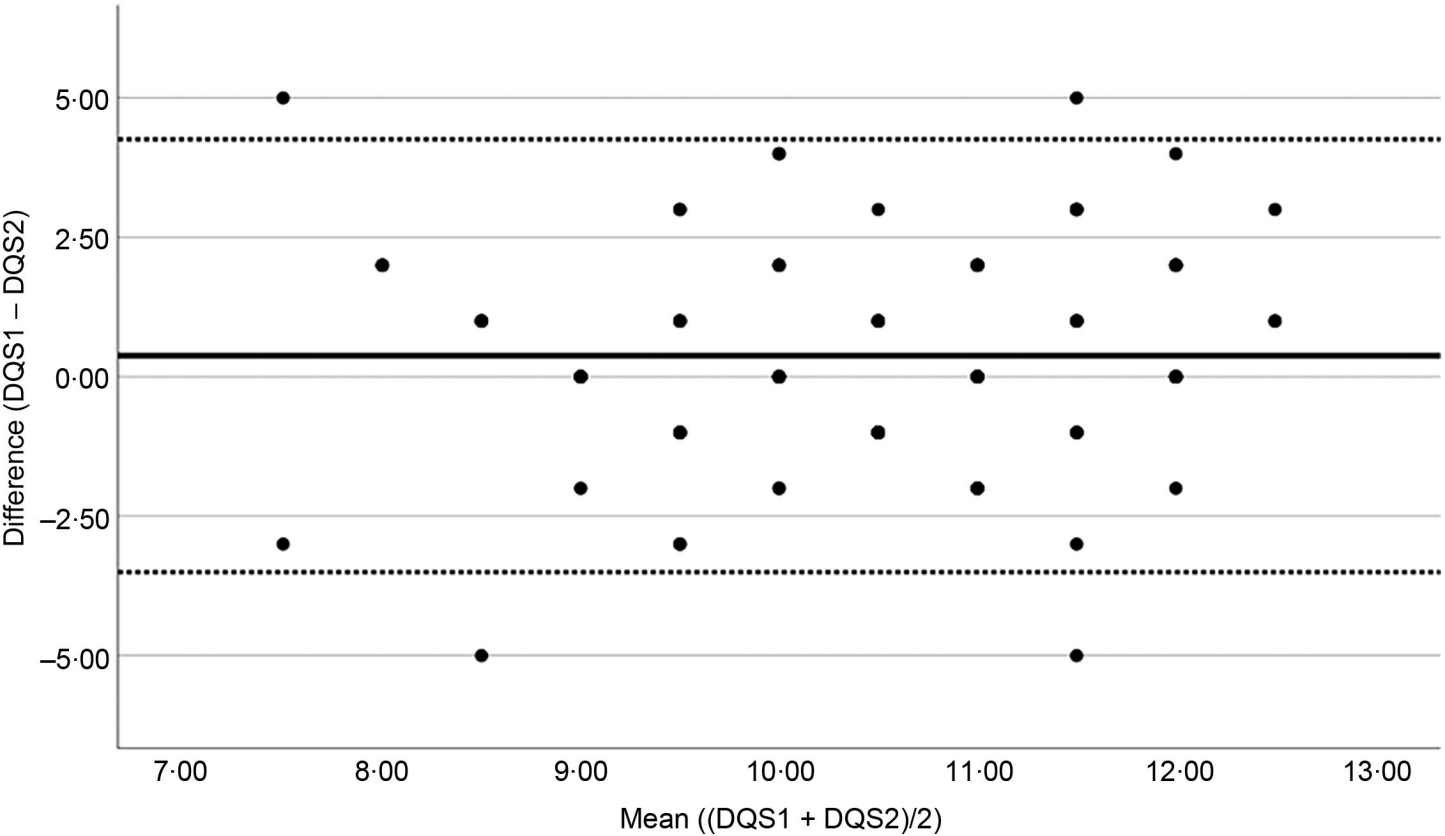
Differences between participant-completed Short Form FFQ (SFFFQ) Dietary Quality Score (DQS_1_) and investigator-completed SFFFQ (DQS_2_) scores plotted against average of DQS_1_ and DQS_2_ (*n* 90). Solid line indicates mean and dotted lines indicated 95 % limits of agreement.

Examination of the Bland Altman plot (Figure 2) shows that the DQS_2_ underestimated the scores of the SFFFQ by a mean difference of 0·4 points compared to the DQS_1_ (95 % limits of agreement: −3·50 to 4·26 points).

## Discussion

Using the described technique in the current study, no statistical differences were observed between the DQS_1_ of the participant-completed SFFFQ_1_ and DQS_2_ generated by the researcher from participant-completed 3dFR (i.e., SFFFQ_2_). At the individual-level, mean difference between DQS_1_ and DQS_2_ was small. The data points were evenly spread indicating no systematic variation for over or under estimation of scores. One concerning factor was the wide spread of the 95 % limits of agreement. The reliability of this technique was supported by the DQS_3_ analysis. Overall, these findings suggest that both dietary assessment instruments captured a similar section of dietary habits at 12–18 weeks’ gestation. The results support the notion that only one dietary assessment was necessary, the 3dFR, to be completed by these participants. Thus, pregnant participant load could be reduced – particularly when time in-clinic is limited – as the 3dFR could be used both to capture short-term nutrient composition and scored for long-term dietary quality and usual intake using the SFFFQ scoring criteria.

When considering if one or both dietary assessment instruments should be used, there are several factors to consider. The SFFFQ, though it has a short administration time, requires participants to recall their dietary intake over the past month or longer, depending on the time frame requirements of the study. Recalling specific dietary intake over a long period of time requires increased attentional resource load and could be challenging for some participants, causing incorrect or incomplete recall which could introduce response or recall bias^(14)^. Additionally, the potential for discrepancy in the reproducibility and validity of use of the SFFFQ during pregnancy, particularly over the course of a long-term follow-up intervention trial, should be noted, especially because FFQ in general are likely to either overestimate or underestimate quantitative food intake over a given period of time^(15,16)^. Furthermore, the semi-quantitative SFFFQ has a limitation in that it is not suitable for estimating absolute dietary intake^(6)^, as the DQS system does not capture the complexity of dietary nutrient composition in the same way as a food intake record analysis using a food analysis software. Additionally, the DQS is not adapted specifically for scoring quality of diet in pregnancy, during which time there may be less food intake variety than when non-pregnant. Interpretation of the individual level score data via a Bland-Altman plot (Figure 2) showed wide limits of agreement, suggesting that there is some slight variation in DQS scores generated from the 3dFR and the SFFFQ. This could be because of the DQS scoring system itself, which could be resolved in future work by optimising the way that the DQS is computed to improve measurement agreement at the individual level for pregnant users.

Another factor to consider when using the SFFFQ, 3dFR, or any other type of dietary assessment instrument is the behaviour change theory that drives the action of recalling or recording one’s dietary intake. Specifically, the act of writing down, logging, or otherwise tracking a behaviour could influence that very behaviour. One framework that may potentially explain this mechanism is the Health Action Process Approach^(17)^, which posits that health behaviours are mediated by the two overarching processes of goal setting and goal pursuit. Under the Health Action Process Approach model, the intention-behaviour relationship is mediated by planning and coping strategies. Monitoring an eating behaviour may affect both the motivational and volitional stages of action^(18)^. Outcome expectancies and recovery self-efficacy could be affected by recalling dietary intake habits during a FFQ, particularly if the recall behaviour is biased due to the social desirability of the individual^(15)^. When used cross-sectionally the effect of the dietary assessment instrument on self-efficacy of the behaviour being assessed may not be significant, but an effect could be elicited with repeated use of the instruments over a longitudinal behaviour change intervention.

When using dietary assessment instruments as part of a nutrition intervention, they can either track the change in behaviour over the course of the intervention or be used as a tool to influence the behaviour change. If the purpose is to track change in overall dietary habit, an FFQ alone would be sufficient. If the purpose is to influence behaviour change, then the act of completing a 3dFR might directly affect one’s feelings of volitional self-efficacy and planning^(18)^. Ideally, an intervention should have dietary assessment instruments that are aligned with the specific goals of the intervention and be used to track adherence to the intervention. If using dietary assessment instruments in this way, appropriate tracking and scoring tools must also be employed to track participant progress of adherence to the specific nutrition goals of the intervention.

To our knowledge, this is the first study to use the SFFFQ and 3dFR in a way to examine short- and long-term dietary assessment challenges among pregnant individuals. Similar work has been conducted in non-pregnant populations^(7,9)^ to elucidate the measurement differences in short-term and long-term dietary assessment instruments. Other methodological strengths were the blinding procedure that was employed and the check for reliability. Though the use of the SFFFQ and 3dFR was a strength of the current investigation, these two instruments were not designed for use solely with pregnant individuals, so future work could focus on designing and/or using pregnancy-specific electronic nutrition assessment instruments. Other limitations include the use of a convenience sample with potential for selection bias and limited generalisability to diverse pregnant populations, as the demographics of the individuals included were mostly Caucasian, had completed education beyond high school and had volunteered to participate in a lifestyle change intervention. The assessment instruments used were self-report, further introducing social desirability or recall bias. Additionally, the method used for multiplying responses about short-term dietary intake has the potential to over- or under-estimate usual intake habits, and assumed low food variety, which may not be representative of actual food intake behaviours.

Several dietary assessment instruments exist for measuring or tracking food intake habits in a research setting. In pregnant populations specifically, the type of instrument used must be carefully chosen to account for changing dietary preferences and intake habits, as well as potentially increased participant fatigue. When comparing the functionality of two commonly used dietary assessment instruments, the SFFFQ scored by pregnant individuals *v*. the SFFFQ scored by the investigator from pregnant individuals’ 3dFR scores, minimal difference in the DQS was observed. Ideally, this technique offers a reasonably accurate representation of dietary habits, especially in the case of nutrition interventions measuring adherence to intervention goals or changes in nutrition habits over time, while also minimising participant burden and recall bias.

## References

[1] Meltzer SJ (2014) Unhealthy lifestyles and gestational diabetes. BMJ (Clin Res ed.) 349, g5549. 10.1136/bmj.g5549 25270049

[2] Walsh JM , McGowan CA , Mahony R , et al. (2012) Low glycaemic index diet in pregnancy to prevent macrosomia (ROLO study): randomised control trial. Br Med J 345, e5605. 22936795 10.1136/bmj.e5605 PMC3431285

[3] Savard C , Lemieux S , Lafrenière J , et al. (2018) Validation of a self-administered web-based 24-hour dietary recall among pregnant women. BMC Pregnancy Childbirth 18, 112. 29685127 10.1186/s12884-018-1741-1 PMC5913813

[4] Bingham SA , Gill C , Welch A , et al. (1997) Validation of dietary assessment methods in the UK arm of EPIC using weighed records, and 24-hour urinary nitrogen and potassium and serum vitamin C and carotenoids as biomarkers. Int J Epidemiol 26, S137–S151. 9126542 10.1093/ije/26.suppl_1.s137

[5] Molag ML , de Vries JH , Ocké MC , et al. (2007) Design characteristics of food frequency questionnaires in relation to their validity. Am J Epidemiol 166, 1468–1478. 17881382 10.1093/aje/kwm236

[6] Cleghorn CL , Harrison RA , Ransley JK , et al. (2016) Can a dietary quality score derived from a short-form FFQ assess dietary quality in UK adult population surveys? Public Health Nutr 19, 2915–2923. 27181696 10.1017/S1368980016001099 PMC10271203

[7] Kirkpatrick SI , Guenther PM , Subar AF , et al. (2022) Using short-term dietary intake data to address research questions related to usual dietary intake among populations and subpopulations: assumptions, statistical techniques, and considerations. J Acad Nutr Diet 122, 1246–1262. 35283362 10.1016/j.jand.2022.03.010

[8] Mottola MF , Giroux I , Gratton R , et al. (2010) Nutrition and exercise prevent excess weight gain in overweight pregnant women. Med Sci Sports Exercise 42, 265–272. 10.1249/MSS.0b013e3181b5419a PMC2886034 20083959

[9] Yang Y , Kim M , Hwang S , et al. (2010) Relative validities of 3-day food records and the food frequency questionnaire. Nutr Res Pract 4, 142–148. 20461203 10.4162/nrp.2010.4.2.142 PMC2867225

[10] Wolfe LA & Mottola MF (2019) PARmed-X for Pregnancy Physical Activity Readiness Medical Examination. Ottawa: Canadian Society for Exercise Physiology. pp. 1–4.

[11] Wall CR , Gammon CS , Bandara DK , et al. (2016) Dietary patterns in pregnancy in New Zealand-influence of maternal socio-demographic, health and lifestyle factors. Nutrients 8, 300. 27213438 10.3390/nu8050300 PMC4882712

[12] Cohen J (1988) Statistical Power Analysis for the Behavioural Sciences, 2nd ed. Hillsdale, NJ: Lawrence Erlbaum Associates, Publishers.

[13] Erdfelder E , Faul F & Buchner A (1996) GPOWER: a general power analysis program. Behavior Res Methods, Instrum Comput 28, 1–11.

[14] Dietary Assessment Primer & Food Frequency Questionnaire. National Institutes of Health, National Cancer Institute (2024). https://dietassessmentprimer.cancer.gov/profiles/questionnaire/index.html (accessed 20 July 2021).

[15] Kristal AR , Peters U & Potter JD (2005) Is it time to abandon the food frequency questionnaire? *Cancer Epidemiol, Biomarkers Prev: A Publ Am Assoc Cancer Res Cosponsored Am Soc Prev Oncol* **14**, 2826–2828. 10.1158/1055-9965.EPI-12-ED1 16364996

[16] Ramage S , McCargar L , Berglund C , et al. (2015) Assessment of pre-pregnancy dietary intake with a food frequency questionnaire in Alberta women. Nutrients 7, 6155–6166. 26225996 10.3390/nu7085277 PMC4555116

[17] Schwarzer R (2016) Health action process approach (HAPA) as a theoretical framework to understand behavior change. Actualidades en Psicología 30, 119–130.

[18] Zhang AC & Downie LE (2019) Preliminary validation of a food frequency questionnaire to assess long-chain *n*-3 fatty acid intake in eye care practice. Nutrients 11, 817. 30978959 10.3390/nu11040817 PMC6521311

